# Pharmacist-guided pre-emptive pharmacogenetic testing in antidepressant therapy (PrePGx): study protocol for an open-label, randomized controlled trial

**DOI:** 10.1186/s13063-021-05724-5

**Published:** 2021-12-14

**Authors:** Céline K. Stäuble, Markus L. Lampert, Samuel Allemann, Martin Hatzinger, Kurt E. Hersberger, Henriette E. Meyer zu Schwabedissen, Christian Imboden, Thorsten Mikoteit

**Affiliations:** 1 Biopharmacy, Department of Pharmaceutical Sciences, University of Basel, Basel, Switzerland; 2 Pharmaceutical Care, Department of Pharmaceutical Sciences, University of Basel, Basel, Switzerland; 3 Institute of Hospital Pharmacy, Solothurner Spitäler AG, Olten, Switzerland; 4 Psychiatric Services Solothurn, Solothurner Spitäler AG and Faculty of Medicine, University of Basel, Solothurn, Switzerland; 5 Private Clinic Wyss, Münchenbuchsee, Switzerland

**Keywords:** Pharmacogenomics, Depression, Antidepressant, Pharmaceutical care, Psychiatry

## Abstract

**Background:**

It is known that only 50% of patients diagnosed with major depressive disorders (MDD) respond to the first-line antidepressant treatment. Accordingly, there is a need to improve response rates to reduce healthcare costs and patient suffering. One approach to increase rates of treatment response might be the integration of pharmacogenetic (PGx) testing to stratify antidepressant drug selection. The goal of PGx assessments is to identify patients who have an increased risk to experience adverse drug reactions or non-response to specific drugs. Especially for antidepressants, there is compiling evidence on PGx influencing drug exposure as well as response.

**Methods:**

This study is an open-label, randomized controlled trial conducted in two study centers in Switzerland: (1) the Psychiatric Clinic of Solothurn and (2) the Private Clinic Wyss in Münchenbuchsee. Adult inpatients diagnosed with a unipolar moderate or severe depressive episode are recruited at clinic admission and are included in the study. If the adjustment to a new antidepressant pharmacotherapy is necessary, the participants are randomized to either Arm A (intervention group) or Arm B (control group). If no new antidepressant pharmacotherapy is introduced the participants will be followed up in an observational arm. The intervention is the service of pharmacist-guided pre-emptive PGx testing to support clinical decision making on antidepressant selection and dosing. As a comparison, in the control group, the antidepressant pharmacotherapy is selected by the treating physician according to current treatment guidelines (standard of care) without the knowledge of PGx test results and support of clinical pharmacists. The primary outcome of this study compares the response rates under antidepressant treatment after 4 weeks between intervention and control arm.

**Discussion:**

The findings from this clinical trial are expected to have a direct impact on inter-professional collaborations for the handling and use of PGx data in psychiatric practice.

**Trial registration:**

ClinicalTrials.govNCT04507555. Registered on August 11, 2020. Swiss National Clinical Trials Portal SNCTP000004015. Registered August 18, 2020.

## Administrative information

Note: the numbers in curly brackets in this protocol refer to SPIRIT checklist item numbers. The order of the items has been modified to group similar items (see http://www.equator-network.org/reporting-guidelines/spirit-2013-statement-defining-standard-protocol-items-for-clinical-trials/).

**Table:** 

Title {1}	Pharmacist-guided pre-emptive pharmacogenetic testing in antidepressant therapy (PrePGx): study protocol for an open-label, randomized controlled trial.
Trial registration {2a and 2b}.	ClinicalTrials.gov, ID: NCT04507555 Swiss National Clinical Trials Portal, ID: SNCTP000004015
Protocol version {3}	Version 3.1, dated 14.09.2021
Funding {4}	Pharmaceutical Care and Biopharmacy Research Groups, University of Basel, 4056 Basel, Switzerland Solothurner Spitäler AG, 4500 Solothurn, Switzerland Privatklinik Wyss AG, 3053 Münchenbuchsee, Switzerland Stiftung zur Förderung des pharmazeutischen Nachwuchses in Basel, 4054 Basel, Switzerland
Author details {5a}	Biopharmacy, Department of Pharmaceutical Sciences, University of Basel, 4056 Basel, Switzerland: Henriette E. Meyer zu Schwabedissen, Céline K. Stäuble Pharmaceutical Care, Department of Pharmaceutical Sciences, University of Basel, 4001 Basel, Switzerland: Samuel Allemann, Kurt E. Hersberger, Markus L. Lampert, Céline K. Stäuble Institute of Hospital Pharmacy, Solothurner Spitäler AG, 4600 Olten, Switzerland: Markus L. Lampert Psychiatric Services Solothurn, Solothurner Spitäler AG and Faculty of Medicine, University of Basel, 4503 Solothurn, Switzerland: Martin Hatzinger, Thorsten Mikoteit Private Clinic Wyss, 3053 Münchenbuchsee, Switzerland: Christian Imboden
Name and contact information for the trial sponsor {5b}	Psychiatrische Dienste Solothurn PD Dr. med. Thorsten Mikoteit Weissensteinstrasse 102 4503 Solothurn Switzerland Phone: +41 32 627 11 11 URL: https://www.solothurnerspitaeler.ch/unsere-spitaeler/psychiatrische-dienste/
Role of sponsor {5c}	This study is an investigator-initiated trial with a sponsor-investigator. The third party funding source had no influence on the study design and will not be involved in its conduct, analysis, and publication of the results.

## Introduction

### Background and rationale {6a}

Successful treatment of depression remains challenging, considering the fact, that only 50% of patients suffering from major depressive disorders respond to the first-line antidepressant treatment [1, 2]. Furthermore, a drug exposure of at least 4 weeks is necessary to assess clinical treatment response [3], possibly making the trial and error approach time-consuming and exhausting for the patient.

It is well known that patients are exhibiting diverse reactions following drug intake. In many cases, inter-individual variability in drug response can be attributed to changes in systemic drug exposure (area under the curve). Meaning the risk of low-drug serum levels resulting in treatment failure and high-drug serum levels leading to toxicity. The organism influences systemic drug exposure by multiple mechanisms namely absorption, distribution, metabolism, and excretion (ADME) of a taken drug molecule. This concept is summarized in the term of pharmacokinetics and comprises a variety of proteins acting on drug molecules in terms of transport (absorption, distribution, and excretion) as well as enzymatic reactions (metabolism). Changes in the activity of the aforementioned proteins will therefore affect systemic exposure and hence drug response. Moreover, the activity of drug transporters and enzymes is influenced by avoidable factors such as drug-drug interactions or drug-food interactions, but also by given predispositions including disease factors and genetics. In fact, a wide range of genes encoding drug transporters and enzymes are polymorphs, occasionally translating into proteins with altered activity [4].

A relevant drug-gene interaction is for example *CYP2D6* with tricyclic antidepressants. The gene encoding for the cytochrome P450 enzyme 2D6 is known to be highly polymorphic including variants that are translated into metabolizing enzymes with increased or reduced activity. Associated phenotypes are termed as ultra-rapid metabolizer or poor metabolizer, respectively [5]. Tricyclic antidepressants are often metabolized via CYP2D6 and have repeatedly been associated with altered pharmacokinetics due to the individual’s genetic predisposition. Based on the rich data on pharmacogenetics, a CPIC (Clinical Pharmacogenetics Implementation Consortium) guideline was published with recommendations for tricyclic antidepressant dosing and for compound selection based on the respective CYP2D6 metabolizer status [6]. Nevertheless, sparse evidence from prospective trials in terms of therapy outcome and cost-effectiveness has so far been an obstacle for implementing *CYP2D6*-guided tricyclic antidepressant prescribing in clinical practice [7].

Remarkably, a wide range of studies is currently being conducted with the aim to identify biomarkers for early and reliable prediction of treatment outcomes of marketed antidepressants, e.g., [8, 9]. However, there is already compiling evidence on pharmacogenetics influencing both, antidepressant exposure and treatment response. This data is gathered and rated according to its level of evidence in the Pharmacogenomics Knowledge Base (PharmGKB) [10]. The aforementioned Clinical Pharmacogenetics Implementation Consortium (CPIC, https://cpicpgx.org/) and the Dutch Pharmacogenetics Working Group (DPWG, https://www.knmp.nl/patientenzorg/medicatiebewaking/farmacogenetica) are publishing guidelines on genotype-guided drug dosing and/or drug selection, which currently includes recommendations for tricyclic antidepressants and selective serotonin reuptake inhibitors. Besides, the Swiss Society for Anxiety and Depression (SGAD) recommends genotyping of *ABCB1* upon antidepressant treatment failure [3]. The latter gene encodes for p-glycoprotein an efflux transporter known for its function in the extrusion of drug molecules and xenobiotics at the blood-brain barrier. Even if not fully validated, it has been hypothesized that patients carrying the wildtype allele of the transporter exhibit increased efflux of substrate antidepressant drugs at the blood-brain barrier, which would translate into decreased drug levels within the central nervous system, and therefore at the place of action. This assumption is based on a limited number of studies, where the *ABCB1* genotype was linked to antidepressant treatment response [11–13].

Even though pharmacogenotyping is not part of routine patient care, pharmaceutical companies cite the known influence of certain polymorphisms on serum levels, adverse drug reactions, and treatment failure in their drug labels [14]. This also applies to several antidepressants authorized in Switzerland namely the tricyclic antidepressants clomipramine, amitriptyline, nortriptyline, and opipramol; the selective serotonin reuptake inhibitors escitalopram, citalopram, fluoxetine, paroxetine, and fluvoxamine; the monoamine oxidase A inhibitor, moclobemide, and the serotonin; and noradrenaline reuptake inhibitors venlafaxine and duloxetine, to name some of them.

Today, pharmacogenetic panel tests are commercially offered. These panel tests consider multiple polymorphic genes involved in pharmacokinetics as well as in the pharmacodynamics of antidepressant drugs. Stratipharm® (humatrix AG, Pfungstadt Germany, https://www.stratipharm.de) is one of the commercial products offering pharmacogenetic panel testing from buccal swabs combined with an evidence-based genotype interpretation. Hitherto, there is only a limited number of prospective clinical studies and to our knowledge non conducted in Switzerland, testing the influence of pre-emptive pharmacogenotyping on patient outcome, whereby limiting the evidence for being advantageous for depression remission or cost-effectiveness over the standard of care, e.g., [15–17]. In fact, pre-emptive panel testing is not yet state of the art in psychiatric practice.

### Objectives {7}

We hypothesize that it is beneficial to incorporate PGx information to guide drug selection and dosing in the treatment of depression, involving clinical pharmacists in processing and evaluating the PGx test results in the context of the individual patient history and current co-medication.

The primary objective of this clinical study is to compare the service of pharmacist-guided PGx testing with the current standard of care for antidepressant selection and dosing with regard to treatment outcome. Accordingly, the following null hypothesis results for the primary endpoint: Therapy response rates after antidepressant treatment for 4 weeks do not differ whether the service of pharmacist-guided pre-emptive pharmacogenetic testing was applied or not.

The secondary objectives are to compare tolerability of the antidepressant pharmacotherapy and overall duration of hospitalization between the intervention and standard care study arms.

### Trial design {8}

This is an open-label, randomized controlled trial, investigating the effectiveness and tolerability of registered antidepressants in adult inpatients with diagnosed major depressive episode.

To prevent selection bias, eligible patients in need of a new antidepressant pharmacotherapy are randomized at the same ratio into either the control or the intervention arm (parallel study arms).

## Methods: Participants, interventions, and outcomes

### Study setting {9}

This is a multicenter clinical trial conducted in Switzerland, at the Psychiatric Clinic of the Solothurner Spitäler AG in Solothurn and the Private Clinic Wyss in Münchenbuchsee.

### Eligibility criteria {10}

Patients are considered eligible for trial inclusion if all of the following criteria are met: (1) ≥ 18 years old, (2) diagnosis of unipolar moderate or severe depressive episode (ICD10: F32.1/32.2/33.1/33.2), and (3) Hamilton Depression Rating Score, version 17 items (HAM-D17) ≥ 17.

If a patient meets any of the following criteria, he or she cannot be included in the trial: (1) acute suicide risk, (2) psychotic symptomatology, (3) other acute serious psychiatric disorder other than depression, (4) excessive consumption of alcohol and/or drugs, (5) severe acute or severe chronic somatic diseases, (6) pregnant or lactating women, and (7) under current treatment with fluoxetine.

### Who will take informed consent? {26a}

The investigators will explain to each participant the nature of the study, its purpose, the procedures involved, the expected duration, the potential risks and benefits, and any discomfort it may entail. Each participant will be informed that the participation in the study is voluntary and that he or she may withdraw from the study at any time and that withdrawal of consent will not affect his or her subsequent medical assistance and treatment. All participants of the study will be provided a participant information sheet and a consent form describing the study and providing sufficient information for participants to make an informed decision about their participation in the study. Participants will be granted enough time to decide whether to participate or not. The formal consent of a participant, using the approved consent form, will be obtained before the participant is submitted to any study procedure. The consent form will be signed and dated by the investigator or his designee at the same time as the participant^1^.

### Additional consent provisions for collection and use of participant data and biological specimens {26b}

With an additional consent form, the patient is asked for permission for further use of the collected biological samples and genetic data, in encrypted form, for not yet further defined future research projects. If the patient consents to the further use of the biological samples and genetic data, the remaining biological material is stored in the Biobank Biopharmazie at the University of Basel, Switzerland.

### Interventions

#### Explanation for the choice of comparators {6b}

The intervention described in the following section {11a} is compared with the current standard of care, where the treating investigator alone selects and doses the antidepressant pharmacotherapy, considering clinical factors only, without taking genetics into account.

#### Intervention description {11a}

The study intervention is the service of pharmacist-guided pre-emptive PGx testing to support clinical decision making for antidepressant selection and dosing.

This service involves genotyping and thereof evidence-based genotype interpretation commercially offered as Stratipharm® (humatrix AG, Pfungstadt Germany, https://www.stratipharm.de). Stratipharm® provides substance-specific recommendations based on current evidence of international guidelines (Clinical Pharmacogenetics Implementation Consortium, CPIC, and Dutch Pharmacogenetics Working Group, DPWG) as well as evidence from clinical studies annotated in the Pharmacogenomics Knowledge Base (PharmGKB, www.pharmgkb.org). Furthermore, clinical pharmacists will process and evaluate the results from PGx testing (Stratipharm®) in the context of the individual patient medication history, medical, and laboratory data (including drug serum levels if available) as well as current co-medication (drug-drug interactions) and forward an individualized recommendation for antidepressant selection and dosing to the treating physician. This intervention is applied pre-emptively, meaning before initiation of a new antidepressant pharmacotherapy during the first week after inclusion into the study.

#### Criteria for discontinuing or modifying allocated interventions {11b}

The intervention under investigation is the service of pharmacist-guided pharmacogenetic testing for the selection and dosing of a new antidepressant pharmacotherapy. Discontinuation or modification of the investigator’s chosen drug and dosage due to any reason is not impaired by the allocated intervention.

#### Strategies to improve adherence to interventions {11c}

The study is conducted on inpatient depression wards of the psychiatric clinics, where monitoring of medication intake and basic laboratory tests (hematology, clinical chemistry, and the like) are part of the routine clinical practice. Furthermore, a clinical study coordinator is supporting the investigators in the conduct of all study-specific assessments, which are internally monitored.

#### Relevant concomitant care permitted or prohibited during the trial {11d}

The introduction of a new antidepressant pharmacotherapy or augmentation strategy (e.g., lithium and the like) are not possible until after randomization when genotyping results are available (7±2 days after inclusion). However, the following measures can be taken in the interim: (1) if an antidepressant therapy has already been taken before entering the clinic, it can be continued during this period, and (2) additional supportive measures are possible, including sleep-promoting pharmacotherapy (e.g., benzodiazepines or low-dose trazodone, mirtazapine, trimipramine).

#### Provisions for post-trial care {30}

After the intervention phase of the study, patients in the control and observation groups as well as their treating physicians will gain access to the PGx data collected.

Study participants do not receive any compensation. However, there are no additional costs for the study participants or the respective health insurance company due to study participation.

In the event of study-related damage or injuries, the liability of the respective institution, Psychiatric Services Solothurn or Private Clinic Wyss, provides compensation, except for claims that arise from misconduct or gross negligence.

#### Outcomes {12}

The primary endpoint is defined as the rate of response to the antidepressant therapy at the end of week 4 of the treatment phase. This time point was chosen based on the recommendations of the SGAD, which advise to assess clinical effectiveness after four weeks of antidepressant pharmacotherapy [3]. Moreover, the response is determined as a reduction in the Hamilton Depression (HAM-D) Scale score of at least 50% from the baseline score [18]. In this study, the 17-item HAM-D questionnaire (HAM-D17) is used.

Secondary endpoints will be assessed to further evaluate the clinical effectiveness of pharmacist-guided PGx testing as an intervention in antidepressant pharmacotherapy. Included are the following endpoints: (1) time to response—time span from the start of antidepressant pharmacotherapy until first assessed response (= HAM-D17 reduction of at least 50% compared to baseline), until end of week 4; (2) remission rate—HAM-D17 score ≤ 8, at week 4; (3) overall change in HAM-D17 score from baseline to end of week 4; (4) time till discharge—time span from admission to discharge from inpatient treatment, assessed up to 3 months; (5) patient depression self-rating—two weekly assessment with Beck-Depression-Inventory (BDI-II) questionnaire [19], until end of week 4; (6) side effect measure—weekly assessment of self-rated frequency, intensity, and burden of side effects (FIBSER score) [20], until end of week 4; and (7) Number of AEs related to antidepressant pharmacotherapy—severity grading ≥ 2 (using CTCAE version 5.0) [21] and causality to antidepressant pharmacotherapy assessed as possible, probable, or definite, until end of week 4.

#### Participant timeline {13}

##### Run-in phase (days -7–0)

**Fig. 1:**
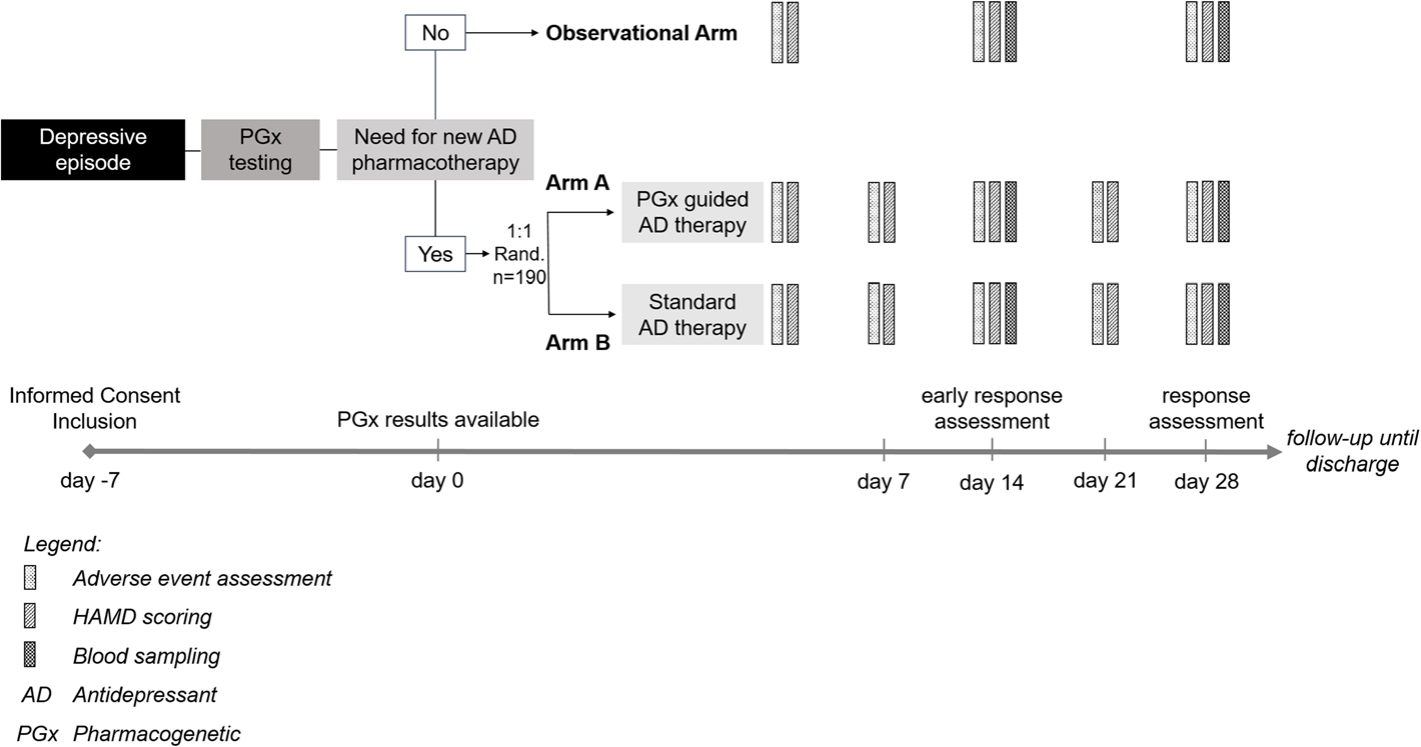
Study procedures

When patients have signed the informed consent and are included in the study, a smear of their oral mucosa is taken and sent to humatrix AG (Pfungstadt Germany, https://www.stratipharm.de) for pharmacogenotyping and phenotype prediction (day -7, see Fig. 1). During the run-in phase, liver and kidney functions will be assessed, taking the following laboratory values: creatinine, eGFR (calculated using CKD-EPI formula), ASAT, ALAT, gamma GT, and total bilirubin. Additional laboratory values will be determined: TSH, C-reactive protein, serum levels of vitamin B12 (total concentration), vitamin D (25-hydroxy-cholecalciferol), and basic hematology (hemoglobin, total erythrocytes, total thrombocytes, total leukocytes). These values will be reassessed at discharge. Moreover, the patient’s antidepressant medication history including the reasons for discontinuation is documented (see Table 1).

**Table 1:** Schedule of assessments

Day	Pre-study	Run-in phase		Treatment phase					Follow-up	Clinic discharge
	−8	−7	−7–0	0	7	14	21	28	35, 42..	
Informed consent for trial participation	**x**									
Eligibility (pregnancy test, drug screening, inclusion, & exclusion criteria)	**x**									
Buccal swab (Stratipharm®)		**x**								
Medical history (previous antidepressant therapies)			**x**							
Lab values (basic hematology, creatinine (eGFR), ASAT, ALAT, total bilirubin, gamma-GT, CRP, TSH, vit. B12, and vit. D)			**x**							**x**
Concomitant medication documentation			**x**	**x**	**x**	**x**	**x**	**x**	**x**	**x**
RANDOMIZATION (only if new antidepressant indicated)				**x**						
PGx pharmaceutical recommendation (only arm A)				**x**						
Start NEW antidepressant				**x**						
HAM-D17	**x**			**x**^**a**^	**x**^**c**^	**x**	**x**^**c**^	**x**	**x**	**x**
AE assessment (antidepressant therapy only)					**x**^**c**^	**x**	**x**^**c**^	**x**	**x**	**x**
FIBSER patient self-assessment				**x**^**a**^	**x**^**c**^	**x**	**x**^**c**^	**x**	**x**	**x**
BDI-II patient self-assessment				**x**^**a**^		**x**		**x**	**x**^**b**^	**x**
Blood sample collection (EDTA and serum)						**x**		**x**		

^a^Baseline scoring before the first intake of new antidepressant

^b^BDI-II assessed in two weekly intervals

^c^Only for study arms A and B

##### Observational arm

If after the run-in phase, at day 0 (±3 days), the adjustment to a new antidepressant pharmacotherapy is evaluated by the treating investigator as not necessary, the patient will be followed up in the observational arm, and the following scores will be assessed on days 0, 14, and 28 ±3 days: (1) HAM-D17, (2) FIBSER, and (3) BDI-II. Additionally, also blood samples will be collected on days 14 and 28 (±3 days) for further genotyping and retrospective assessment of serum drug concentrations (see Fig. 1). After day 28 of the study, the already collected genotyping data will be interpreted by a clinical pharmacist and made accessible to the treating physician.

##### Randomization and procedures for arms A and B

If after the run-in phase, at day 0 (±3 days), the treating physician assesses the adjustment to a new antidepressant pharmacotherapy as necessary, and the patient is randomized to either arm A (intervention group) or arm B (control group) (see Fig. 1).

In arm B, the treating physician alone, according to the current standard of care considering clinical factors only, will determine the selection and dosing of the new antidepressant pharmacotherapy. The treating physician and patient will be blinded to the results of the previously conducted pharmacogenotyping for patients randomized to arm B until day 28. For patients in arm B, results from prior pharmacogenetic testing will be interpreted by a clinical pharmacist and made accessible to the treating physician after day 28.

In arm A, a clinical pharmacist will process and evaluate the results from PGx testing (Stratipharm®) in context of the individual patient history as well as current co-medication and forward an individualized recommendation for antidepressant selection and dosing to the treating physician at day 0.

In both arms, the newly prescribed antidepressant pharmacotherapy intake is continuously documented and therapy response observed over a period of 28 days with weekly assessments of adverse events related to the antidepressant medication (using CTCAE version 5.0) and scorings of HAM-D17 (days 0, 7, 14, 21, and 28; ±3 days) as well as patient self-assessments of FIBSER score (days 0, 7, 14, 21, and 28; ±3 days) and BDI-II score (days 0, 14, and 28; ±3 days) (see Table 1).

If patients in either study group remain in the clinic after day 28 (±3 days), a weekly follow-up of HAM-D17, FIBSER, and two-weekly BDI-II are continued until discharge (see Table 1).

#### Sample size {14}

The sample size was calculated to be 95 patients per study arms A and B. This was done taking into account the following criteria: power = 80%, *α* = 5%, response rate standard care = 0.5 [1, 2] and response rate PGx guided = 0.7 [16]. For the observational study arm, there is no sample size calculation needed, since this arm does not contribute to the primary endpoint.

#### Recruitment {15}

Participants are recruited and screened for eligibility by the treating investigator in daily clinical practice, during the regular hospital admission interview, when entering the clinic for an inpatient stay.

### Assignment of interventions: allocation

#### Sequence generation {16a}

Allocation of participants to study arms A or B is based on a computer-generated allocation sequence without any stratifying factors (static unstratified multi-block randomization).

#### Concealment mechanism {16b}

Randomization for participant allocation into study arms A and B is performed by the appointed clinical pharmacist without knowledge of the allocation sequence, within the web-based electronic data capture program secuTrial® (interactive Systems GmbH, Berlin, Germany). This is an open-label study; however, the service of individual processing and evaluation of the genotyping data is only conducted by the respective clinical pharmacist for participants allocated to arm A (intervention arm) at baseline (day 0). To further guarantee blinding to the genotyping results, assessed during the run-in phase, only the appointed clinical pharmacists do have password-protected access to the genetic data from Stratipharm®.

#### Implementation {16c}

Separated departments conduct each process of generating the allocation sequence, enrolling the participants, and assigning participants to interventions. The allocation sequence is implemented by an independent data management team of the clinical trials unit at the University of Basel in Basel, Switzerland. The investigators at the Psychiatric Clinic in Solothurn and Private Plinic Wyss in Münchenbuchsee, in Switzerland, conduct the enrolment of participants. Furthermore, the responsible clinical pharmacists of the Solothurner Spitäler AG association perform randomization electronically.

### Assignment of interventions: blinding

#### Who will be blinded {17a}

This is an open-label study, hence no blinding of intervention allocation is possible and needed. However, the genetic data assessed during the run-in phase will only be shared with the treating physician and patients allocated to the control or observational group after day 28 of the treatment phase.

#### Procedure for unblinding if needed {17b}

Not applicable, as described in section {17a}, this is an open-label trial.

### Data collection and management

#### Plans for assessment and collection of outcomes {18a}

Outcomes described in section {12} are assessed and collected using validated questionnaires and scoring tools (HAM-D17, FIBSER, BDI-II). FIBSER and BDI-II are patient-self-rated scores where the patients fill the according questionnaires independently [19, 20]. The HAM-D17 score is assessed by a trained rater (e.g., treating physician or specifically designated personnel) [18] who is however, not blinded to the patient allocation. The treating physician does the antidepressant adverse event assessment and grading according to the CTCAE version 5.0 [21]. The described data is collected on paper forms approved by the local ethics committee or directly entered into the patient electronic medical record when appropriate.

#### Plans to promote participant retention and complete follow-up {18b}

Assessments and follow-ups are only conducted during the participants’ inpatient stay at the psychiatric clinic. Therefore, the risk of loss to follow-up and deviation to the protocol due to failure to comply with the study visits are considered negligible. However, patients who withdraw their consent (e.g., refuse further data collection) will be informed that all data collected until the time point of their withdrawal will be kept coded and used for analysis.

#### Data management {19}

The data collected on paper forms or in the patient electronic medical record are regularly transferred to an approved electronic database by a designated clinical research coordinator. The data will be coded with the according patient identifier, once transferred to the electronic database.

Study personnel will be trained on all important study-related aspects. After inclusion and trial participation of the first patient and regularly thereafter, the quality and accuracy of data collection will be checked internally.

All study data are archived for 10 years after study termination or premature termination of the study. The source data and all trial material will be stored in the archive of the study clinic.

#### Confidentiality {27}

Trial and participant data will be handled with uttermost discretion and is only accessible to authorized personnel who require the data to fulfill their duties within the scope of the study. On the CRFs and other study-specific documents, participants are only identified by a unique participant number^2^. The participant identification list is kept in a locked place under the supervision of the principal investigator at the study site. Only encrypted data, which cannot be traced back to the individual study participant without knowledge of the identification list, will ever leave the study site. Furthermore, all collected data is stored on password and safety-back-up protected drives of the study clinic, which can only be accessed by authorized personnel. However, the data gathered is always traceable to the source data (e.g., patient medical records or questionnaires) at the study site, based on the accordingly documented patient identifier and the original collection date.

For quality assurance the sponsor, the Ethics Committee or an independent trial monitor may visit the research sites. Direct access to the source data and all study-related files is granted on such occasions. All involved parties keep the participant data strictly confidential^3^.

#### Plans for collection, laboratory evaluation, and storage of biological specimens for genetic or molecular analysis in this trial/future use {33}

Collected oral mucosa on day -7, for pharmacogenotyping at humatrix AG in Germany (Stratipharm®), is destroyed 3 weeks after completion of the analysis and only the genetic data remain. These are encrypted with the sample number for evaluation and thus assigned to a database (server without internet access). Only designated employees have access to the data and must comply with strict data protection regulations. The genetic data will be kept until withdrawal. The laboratory in Germany has standards equivalent to those in Switzerland. Results of the pharmacogenetic testing by Stratipharm are made available for the responsible clinical pharmacist at the study site through a web portal, which is password protected and therefore only accessible by authorized personnel. The genetic data assessed by Stratipharm will be coded with the according patient identifier once transferred to the CRF and archived after study termination for at least 10 years.

Collected blood samples, 4 ml EDTA whole blood, and 6 ml serum on days 14 and 28 are not identified by participant name but by a unique participant identifier. For processing and further analysis, the biological material is transferred using dry ice shipment when necessary, to the biobank Biopharmazie at the University of Basel. There, the blood samples and thereof isolated DNA and processed blood serum are appropriately stored between −20 and −80°C in a restricted area only accessible to authorized personnel. Laboratory personnel handling the biological material outside of the study clinic do not have access to the patient identification list and therefore cannot trace the samples back to the individual participants. The planned analysis includes further pharmacogenetic targets and substance blood concentrations using standardized and established methods (e.g., TaqMan® genotyping assays, direct DNA Sanger sequencing, and LC-MS/MS or HPLC). The results of these examinations are not taken into account for the study treatment decisions and will only be assessed after day 28.

The collected blood samples and thereof processed samples (DNA and blood serum) will be stored in the biobank Biopharmazie at the University of Basel until study publication or early study termination. However, if participants consent to the further use of the assessed and encrypted genetic data and biological material, these will be stored over a yet undetermined time period and for a yet undetermined use in the study clinic or in the Biobank Biopharmazie respectively.

### Statistical methods

#### Statistical methods for primary and secondary outcomes {20a}

The primary outcome of this study is the response rate at day 28 in study arms A and B. The response is defined as a reduction of the HAM-D17 score of at least 50% compared to baseline at day 0. Additionally, the following secondary outcomes are assessed: time to response, remission rate, overall change in HAM-D17 score, laytime in the clinic, change in BDI-II score, change in FIBSER score, and number of AEs related to antidepressant pharmacotherapy. For statistical analyses, the software packages of “IBM SPSS Statistics” and “GraphPad Software” are used. A descriptive statistics analysis for all variables is performed. Fisher’s exact test or *t* test is used to compare pairwise differences between groups and between baseline and follow-up visits as per data type. To measure the correlation between variables, the Spearman coefficient will be used. Significance level is two-sided, *α* = 0.05. For further statistical analyses, adjustments for confounding factors will be taken into account. Any deviation from the original statistical plan will be described and justified in the final trial report.

#### Interim analyses {21b}

Interim analyses are possible, according to time points that are not previously defined. If an interim analysis reveals an undue disadvantage of PGx intervention (e.g., increased number of adverse events) compared to the standard care group, the study will be stopped.

#### Methods for additional analyses (e.g., subgroup analyses) {20b}

Currently, there are no subgroups or adjusted analyses planned. However, the stored samples allow the assessment of additional genetic biomarkers, which may be used for further stratification of the patient cohort and therefore subgroup analyses.

#### Methods in analysis to handle protocol non-adherence and any statistical methods to handle missing data {20c}

Missing data will be retrospectively retrieved from medical records if possible. If the missing data cannot be retrieved, the last observed value will be used for analysis. Study drop-outs are replaced to achieve the final calculated study size of 95 patients per study arms A and B.

#### Plans to give access to the full protocol, participant level-data, and statistical code {31c}

To grant public access to the study procedure and status, it is registered and updated whenever necessary in the Swiss National Clinical trial Portal (SNCTP000004015) and the ClinicalTrials.gov register (NCT04507555), of which the latter is listed in the WHO International Clinical Trials Registry Platform (ICTRP; http://www.who.int/ictrp/en/).

### Oversight and monitoring

#### Composition of the coordinating center and trial steering committee {5d}

This trial is an investigator-initiated multicenter clinical study. There is no external coordinating center or trial steering committee involved.

#### Composition of the data monitoring committee, its role, and reporting structure {21a}

The local ethics committee classified this trial as low risk. Therefore, internal monitoring by designated personnel is applicable. Internal monitoring is performed after the inclusion of the first participant and after study termination. In between internal monitoring will be applied as needed. The accuracy and completeness of the transfer of data from the original source to the CRF as well as completeness and storage of blood samples are checked. Study relevant source data and documents are accessible to internal monitors and in any case to external auditing. Questions are answered during monitoring and auditing. An internal data monitoring plan has been approved by the local ethics committee prior to study initiation.

#### Adverse event reporting and harms {22}

The intervention under investigation consists of a clinical pharmacist’s service resulting in a recommendation of an antidepressant drug approved in Switzerland. Study procedures include taking a swab of the oral mucosa and drawing blood samples for further analysis. These procedures are considered low-risk and routine clinical practice. Only AEs related to the antidepressant pharmacotherapy are reported, since this is a secondary outcome of the study.

#### Frequency and plans for auditing trial conduct {23}

External auditing by the local ethics committee is possible at any time of the study conduct but not planned and communicated in advance.

#### Plans for communicating important protocol amendments to relevant parties (e.g., trial participants, ethical committees) {25}

Substantial changes to the study setup and study organization, the protocol, and relevant study documents are submitted to the local ethics committee for approval before implementation. Under emergency circumstances, deviations from the protocol to protect the rights, safety, and well-being of human subjects may proceed without prior approval of the EC. Such deviations shall be documented and reported to the Ethics Committee as soon as possible^4^. Patients still involved in the study conduct are asked to reconsent in case of a substantial amendment concerning the study procedures. Any non-substantial amendments are communicated to the EC in an annual report.

#### Dissemination plans {31a}

The investigators will publish and will make the study results available to the public in peer-reviewed journals. Besides, our findings will be communicated during national and international congresses relevant to clinicians and academics of associated fields.

## Discussion

Despite the growing evidence already incorporated in international pharmacogenomics guidelines, PGx testing for antidepressant selection and dosing is not yet part of routine psychiatric practice. An important reason for this is the sparse number of prospective clinical trials, thereby limiting the evidence for the potential advantage over the current standard of care approach in antidepressant prescribing. Furthermore, in Switzerland, PGx testing requires prescribing by a pharmacologist, to ensure health insurance coverage. Pharmacologists however are not routinely involved in psychiatric clinics and are therefore hard to reach out to in daily practice. Another very likely reason that prevents psychiatrists from incorporating PGx information into their prescribing habits may include missing established procedures and in general a lack of resources in psychiatric clinics to substantially enable individualized PGx information processing to support drug selection and dosing. Therefore, an inter-professional collaboration between psychiatrists and clinical pharmacists may be of benefit for the treatment and provide a supportive framework for an individual interpretation and use of PGx data [22]. Independent of PGx, this interdisciplinary approach has been studied before and was found to have a positive impact on identifying drug-related problems [23]. Moreover, psychiatrists have been reported to mainly seek help from involved pharmacists in terms of drug selection [24]. As described beforehand, PGx information might add substantial value in answering this question. Therefore, the goal is to investigate the service of pharmacist-guided pre-emptive PGx testing in antidepressant therapy.

In the herein described clinical trial, the intervention in question (pharmacist-guided pre-emptive PGx testing) is compared to the current standard of care approach in antidepressant selection and dosing in an open-label, parallel-arm, randomized trial. This trial design allows direct comparison of the two approaches, minimizing selection bias by randomly assigning patients to either intervention or control group. Furthermore, the trial design was intended to fit as naturally as possible into the clinic’s daily routine, enabling direct transfer of any study results into practice. However, the pragmatic setup of the trial does not allow blinding of the treating physician nor the patient on group allocation. Another limitation is that the field of pharmacogenetics is constantly evolving, with new findings resulting in further stratification of the recommendations published by CPIC, DPWG, and PharmGKB. Within the study, we will use the drug-genotype interpretation of Stratipharm®, which is based on the aforementioned sources. Therefore, a certain change in recommendations from beginning to end of the clinical trial cannot be excluded. Furthermore, we cannot rule out a potential training effect of the involved physicians, which may lead to favoring the prescription of antidepressants without PGx implications. It should also be emphasized that this trial investigates the effect of an integrated approach to clinical pharmaceutical consulting, of which pharmacogenetic data are a part and cannot be evaluated in isolation. Nevertheless, this trial entails only minimal risks comparable to routine clinical procedures. These relatively minimal risks face a high expected gain of knowledge and evaluation of potential benefits for future patients. In summary, we expect this trial to have a direct impact on routine psychiatry and pharmacy practice.

### Trial status

Protocol version number and date: Version 3.1, September 14, 2021.

Date recruitment began: September 15, 2020.

Date recruitment approximately will be completed: September 14, 2023.

## Data Availability

Investigators will have full access to the final trial dataset.

## Abbreviations

AE: Adverse event
BDI-II: Beck depression-inventory II
CPIC: Clinical Pharmacogenetics Implementation Consortium
CRF: Case report form
CTCAE: Common Terminology Criteria for Adverse Events
CYP: Cytochrome P450
DPWG: Dutch Pharmacogenetics Working Group
eCRF: Electronic case report form
FIBSER: Frequency, Intensity, and Burden of Side Effects Rating
GCP: Good Clinical Practice
HAM-D: Hamilton rating scale for depression
MDD: Major depressive disorder
PGx: Pharmacogenetic
PharmGKB: Pharmacogenomics Knowledge Base
SGAD: Swiss Society for Anxiety and Depression

## Footnotes

1 Binding wording by the Swiss Association of Research Ethics Committees - Clinical Protocol Template for ClinO, Chapter 4 »Other Clinical Trials«, Version 1.0, 30.08.2018

2 Binding wording by the Swiss Association of Research Ethics Committees - Clinical Protocol Template for ClinO, Chapter 4 »Other Clinical Trials«, Version 1.0, 30.08.2018

3 Binding wording by the Swiss Association of Research Ethics Committees - Clinical Protocol Template for ClinO, Chapter 4 »Other Clinical Trials«, Version 1.0, 30.08.2018

4 Binding wording by the Swiss Association of Research Ethics Committees - Clinical Protocol Template for ClinO, Chapter 4 »Other Clinical Trials«, Version 1.0, 30.08.2018
